# Prevalence of metabolic syndrome in primary health settings in Qatar: a cross sectional study

**DOI:** 10.1186/s12889-020-08609-5

**Published:** 2020-05-03

**Authors:** Mohamed Ahmed Syed, Ahmed Sameer Al Nuaimi, Abdul Jaleel A. Latif Zainel, Hamda Abdulla A/Qotba

**Affiliations:** Directorate of Clinical Affairs, Primary Health Care Corporation, P.O. Box 26555, Doha, Qatar

**Keywords:** Metabolic syndrome, Primary care, Qatar, Obesity, Hypertension, Dyslipidaemia diabetes, Prevalence

## Abstract

**Background:**

In Qatar, prevalence of metabolic components is significantly higher compared to other countries. It is therefore urgent to understand the prevalence of metabolic syndrome (MetS) with the goal of identifying etiologic factors in Qatar. This study was undertaken to estimate the prevalence of MetS, by age, gender and nationality within primary care settings in Qatar. In addition, it determined the independent effects of risk factors on the prevalence of MetS.

**Methods:**

A cross-sectional study design was used. Data for individuals aged ≥18 and who visited a publicly funded primary health centre in Qatar during 2017 were extracted from electronic medical records and analysed.

**Results:**

The findings showed that the prevalence of individual MetS components ranged between 48.5–60.3%. Overall prevalence of MetS was 48.8% (*N* = 62,492) in the study population. Prevalence of MetS increased with age. 50.3% of the population within the 40–49 year age group had MetS. In this age band, individuals were 5.1 times more likely of having MetS compared to the 18–29 year age group. MetS was slightly more prevalent in men (56 .7%) compared to women (42.5%). However, men were 1.33 times more likely of having MetS compared to women. The prevalence of MetS ranged between 20.6 - 60% across nationalities. It was most prevalent in Southern Asians (60%), followed by Northern Africans (50.7%) and Western Asians (excluding Qatar) (46.8%). Prevalence of MetS in Qataris was 43%. Southern Asians, Northern African and Western Asians were 1.73, 1.38 and 1.17 more likely to have MetS compared to Qataris.

**Conclusions:**

The study provides essential epidemiological information required by decision makers. Although not nationally representative, this study is suggestive of a higher prevalence of MetS among a younger population, men and in Southern Asian, Northern African and Western Asian nationalities. Prevention, treatment and control of MetS is a public health problem in Qatar. More studies are needed to establish which public health interventions are likely to be effective in Qatar.

## Background

Metabolic syndrome (MetS) is a complex asymptomatic, pathophysiological state [1]. It is defined by the co-occurrence of insulin resistance, obesity, atherogenic dyslipidemia and hypertension [2]. It is generally agreed that having three or more of these aetiologically linked cardiometabolic risk factors increases the risk of developing multiple chronic diseases (such as cardiovascular disease, type 2 diabetes (T2DM), arthritis, chronic kidney disease, schizophrenia, several types of cancer and of early death [1–14] .

During the past several decades, the prevalence of MetS has markedly increased worldwide [15]. It is estimated that 25% of the world’s population has MetS [16] although this estimate varies widely due to the age, ethnicity, and gender of the population studied [17]. MetS is associated with high socioeconomic cost [18]. Behavioral and environmental changes, such as adoption of a westernized diet and sedentary lifestyle following the socioeconomic rise in developing countries, are thought to be the main reasons for this pandemic of metabolic syndrome [19]. Qatar, a peninsular Arab country with a backed by the world’s third-largest natural gas and oil reserves, has experienced a rapid socioeconomic growth during the last two decades and the income of its residents has been rising.

In Qatar, prevalence of MetS components is significantly higher compared to other countries. It is estimated 23% of Qataris and 18.3% non-Qataris have T2DM [20]. Prevalence of obesity rates in Qatar ranks fifth globally and is estimated to be 44% in men and 54.7% in women [21]. Similarly, in Qatar the prevalence of hypertension and raised total cholesterol was reported as 32.9 and 21.9% respectively. Based on existing trends, substantial increases in the proportion of the population is likely to meet MetS criteria thus at an increased risk for more serious chronic conditions and premature death.

Studies have previously been conducted to establish prevalence and determinants of metabolic syndrome provide valuable information, however, they include Qataris only [22]. Given expatriates account for 88% of Qatar’s population [23], more recent studies which also include them are required. It is therefore urgent to understand the MetS prevalence with the goal of identifying etiologic factors in Qatar. Early identification of MetS components could lead to targeted public health interventions to prevent the development of the syndrome, and thus chronic disease risk in later life. This study was undertaken to estimate the prevalence of MetS, by age, gender and nationality within primary care settings in Qatar. In addition, it determined the independent effects of risk factors on the prevalence of metabolic syndrome.

## Methods

### Study settings

Qatar has been investing significantly in its healthcare system. This includes a universal publicly funded primary healthcare service delivered by the Primary Health Care Corporation (PHCC). PHCC is the largest primary care provider in the country publicly with 27 health centres (all accredited by Accreditation Canada International and distributed across three geographical regions).

### Study population and data collection

The study population includes both Qataris and non-Qataris registered at a PHCC health centre, aged ≥18 and who visited a health centre between 1 January 2017 and 31 December 2017. Demographic and diagnosis data were extracted from the electronic medical records (EMR) for the defined population.

### Definitions and data analysis

A total of 421,283 individuals accessed primary healthcare services in 2017. The National Cholesterol Education Program Adult Treatment Panel III (NCEP ATP III) component definitions was adapted for the study [24] (Supplementary file; Table A) based on the data available in the EMR system. As with NCEP ATP III, MetS is considered present if three or more of the following five criteria are met (or medication was taken to control them).

The proportion of least and most missing data for a MetS component was high blood pressure (3.3%) and low serum high-density lipoprotein (HDL) (56.4%) respectively (Supplementary file; Table B). Of these 127,941 had data available for all five MetS components (Supplementary file; Table C). This data was used for the study.

All data were analysed using the ‘Statistical Package for the Social Sciences’ statistical software package. Basic descriptive statistics were used to analyse the population components (age, gender and nationality; see supplementary file for classification) and MetS components.

A multiple logistic regression model with the risk of having MetS as the dependent (outcome) variable and age, gender and nationality as predictor (explanatory) variables was developed. Crude age specific and age adjusted prevalence rates for MetS components by nationality were calculated. Age-adjusted prevalence rates were calculated using the World Health Organisation (WHO) World Standard Population (2000–2025) to allow comparisons [25].

### Ethical considerations

The study presented a minimal risk of harm to its subjects, and the data collected for it were anonymised. None of the subjects’ personal information was available to the research team. Overall, the study was conducted with integrity according to generally accepted ethical principles and was approved by the PHCC’s independent ethics committee (PHCC/RS/18/02/003).

## Results

### Population components

MetS component data was available for 127,941 individuals who accessed primary healthcare services in 2017 (Table 1). Individuals in the 40–49 year age group accounted for approximately 24% of the population. 55.4% of the study population was women. 35.8% of the population was Qatari and 64.2% non-Qatari. The largest non-Qatari nationalities were represented by Southern Asian (25.2%), Western Asian (15.2%) and Northern African (16.7%) - They accounted for 57.1% of the total study population.

**Table 1:** Population components of patients

Population Demographics	Population with all 5 MetS component data available (Total population = 127,941)	
	N	%
**Age (years)**		
18–29	15,744	12.3
30–39	28,832	22.5
40–49	31,650	24.7
50–59	28,948	22.6
60+	22,767	17.8
**Gender**		
Women	70,853	55.4
Men	57,088	44.6
**Nationality**		
Qatar	45,854	35.8
Southern Asia	32,205	25.2
Western Asia (excluding Qatar)	19,431	15.2
Northern Africa	21,397	16.7
South-eastern Asia	5447	4.3
Sub-Saharan Africa	1662	1.3
Northern America	788	0.6
Northern Europe	548	0.4
Latin America and the Caribbean	85	0.1
Eastern Europe	126	0.1
Southern Europe	126	0.1
Western Europe	116	0.1
Australasia	95	0.1
Eastern-Central Asia	59	0.0

### Prevalence of MetS components

The overall prevalence of metabolic syndrome was 48.8% (N = 62,492) in the study population. The prevalence of individuals MetS components ranged between 48.5–60.3% (Table 2)

**Table 2:** Prevalence of MetS components in patients for whom all 5 MetS component data was available

Metabolic Syndrome components (Total population = 127,941)	N	%
Insulin Resistance (Serum Fasting Glucose> = 100 mg/dl or HbA1c > =5.56) or T2DM	77,100	60.3
High Blood Pressure (Systolic or Diastolic) or hypertensive	67,758	53.0
Obesity (Body Mass Index > = 30 kg/m2 or central obesity)	63,315	49.5
Low serum HDL (< 1.04 mmol/L in male and < 1.3 mm in female)	62,042	48.5
**Metabolic syndrome (at least 3 components present)**	**62,496**	**48.8**

**Fig. 1:**
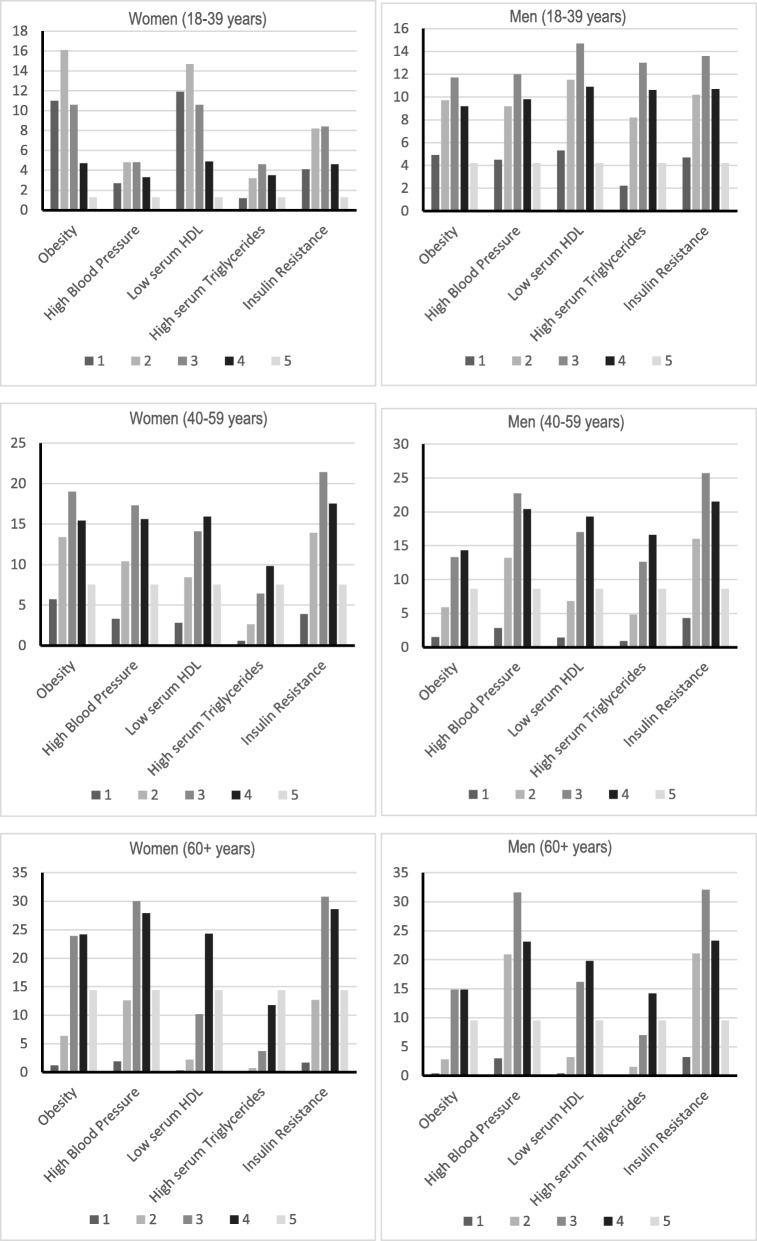
Prevalence of having one, two, three, four, or five metabolic syndrome component by age and gender

The prevalence of each metabolic syndrome component combination and the prevalence of having one, two, three, four, or five metabolic syndrome component by age and gender are shown in Fig. 1. In younger adults (18–39 years), the most prevalent MetS components were obesity (16.1%) and low HDL (14.7%) in combination with another MetS component in women while low HDL (14.7%) and insulin resistance (13.6%) in combination with two other MetS component were most prevalent in men. In middle aged adults (40–59 years), insulin resistance (21. 4%) and obesity (19%) in combination with two other MetS components were most prevalent in women while insulin resistance (25. 7%) and high blood pressure (22.7 5) in combination with two other MetS components were most prevalent in men. In older adults (60 + years), insulin resistance and high blood pressure in combination with two other MetS components were most prevalent in women (30. 8 and 30% respectively) and men (32.2 and 31.6% respectively).

### Prevalence of MetS by age, gender and nationality

Prevalence of MetS increased with age. 50.3% of the population within the 40–49 year age group had MetS (Table 3). They were 5.1 times more likely of having MetS compared to the 18–29 year age group (Table 4). MetS was slightly more prevalent in men (56 .7%) compared to women (42.5%). However, men were 1.33 times more likely of having MetS compared to women (Table 4).

**Table 3:** Population components of patients attending publicly funded primary health centers in 2017 with all five MetS component data available

Population Demographics	Prevalence of MetS (Total population = 127,941)	
	N	%
**Age (years)**		
18–29	2349	14.9
30–39	9362	32.5
40–49	15,906	50.3
50–59	18,592	64.2
60 +	16,287	71.5
**Gender**		
Women	30,145	42.5
Men	32,351	56.7
**Nationality**		
Southern Asia	19,310	60
Northern Africa	10,853	50.7
Western Asia (excluding Qatar)	9090	46.8
Australasia	41	43.2
Qatar	19,707	43
Northern America	333	42.3
South-eastern Asia	2204	40.5
Northern Europe	210	38.3
Sub-Saharan Africa	600	36.1
Latin America and the Caribbean	30	35.3
Eastern-Central Asia	20	33.9
Eastern Europe	37	29.4
Western Europe	34	29.3
Southern Europe	26	20.6

**Table 4:** A multiple logistic regression model with the risk of having metabolic syndrome and age, gender and nationality as predictors

	Adjusted OR	P
**Age (years)**		
30–39 compared to 18–29	2.45	< 0.001
40–49 years old compared to 18–29	5.10	< 0.001
50–59 years old compared to 18–29	9.12	< 0.001
60+ years old compared to 18–29	13.11	< 0.001
**Gender**		
Men compared to women	1.33	< 0.001
**Nationality**		
Northern Africa compared to Qatari	1.38	< 0.001
Sub-Saharan Africa compared to Qatari	0.81	< 0.001
Latin America and the Caribbean compared to Qatari	0.66	0.08[NS]
Northern America compared to Qatari	0.67	< 0.001
Eastern-Central Asia compared to Qatari	0.64	0.13[NS]
South-eastern Asia compared to Qatari	0.93	0.018
Southern Asia compared to Qatari	1.73	< 0.001
Western Asia (excluding Qatar) compared to Qatari	1.17	< 0.001
Eastern Europe compared to Qatari	0.52	0.002
Northern Europe compared to Qatari	0.68	< 0.001
Southern Europe compared to Qatari	0.31	< 0.001
Western Europe compared to Qatari	0.44	< 0.001
Australasia compared to Qatari	0.63	0.035

The prevalence of MetS ranged between 20.6 - 60% across nationalities. It was most prevalent in Southern Asians (60%), followed by Northern Africans (50.7%) and Western Asians (excluding Qatar) (46.8%). Prevalence of MetS in Qataris was 43% (Table 3). Southern Asians, Northern African and Western Asians were 1.73, 1.38 and 1.17 more likely to have MetS compared to Qataris (Table 4).

Overall age adjusted MetS prevalence across nationalities was 421/1000 (see supplementary file for MetS prevalence across nationalities). The highest age adjusted rates were 507/1000, 450/1000 and 409/1000 seen in Southern Asians, Northern African and Western Asians (excluding Qatar) respectively. The lowest age adjusted rate was 181/1000 in Southern Europeans. Southern Asians have an age adjusted prevalence are 2.8 times higher than Southern Europeans.

## Discussion

The prevalence of metabolic syndrome (MetS) is on the rise worldwide [26]. This study is potentially the first comprehensive study describing the prevalence of MetS which includes both Qatari and non-Qatari populations in publicly funded primary care settings in Qatar. The study found prevalence of MetS was 48.8%. It also found that over 50% of the study population had a prevalence of insulin resistance or diagnosis of T2DM. A meta-analysis reported a pooled estimate prevalence of MetS in the Middle East as 25% [27]. This highlights the significant burden of MetS in the country.

This study, as in all epidemiologic studies, found the prevalence of MetS to increase with age [28]. This is not surprising since there are many commonalities in biochemical changes of aging process and metabolic syndrome/diabetes [28]. However it is worth noting that the prevalence in younger age groups was much higher compared to that reported in other studies. A European study reported a prevalence of approximately 10, 20 and 38% in 19–39, 40–49 and 60–78 year age ranges respectively [29] while a prevalence of 23, 50.3% and 71. 5% was found in this study. The onset of MetS in the Qatari population is approximately twice as early compared to European populations. These findings suggests a need for efforts to increase prevention strategies early, ideally when any one of the MetS components manifests and before development of all three required for the formal definition of metabolic syndrome.

When comparing prevalence of MetS by gender, men (56.7%) had a higher prevalence compared to women (42.5%) in this study. MetS was 1.33 times more prevalent in men compared to women. These findings are in line with a study that reported on the prevalence of non-communicable diseases (NCDs) in primary care settings in Qatar. NCDs, which are outcomes of MetS components, were more prevalent in men compared to women [20]. However in the European population, MetS was more prevalent in women (32.1%) compared to men (19.9%) [29]. Similarly, a systematic review reported a higher prevalence of MetS in women (32.1 to 42.7%) compared to men (20.7 to 37.2%) in Gulf Cooperative Council countries (Bahrain, Kuwait, Oman, Qatar, Saudi Arabia and the United Arab Emirates) [30].

The current study found a higher prevalence rate of 43% (373/1000) amongst Qataris compared to previous studies (26.5–33.7%) [22, 31]. While Southern Asian (60%, 507/1000), Northern African (50.7%, 450/1000) and Western Asians (46.8%, 409/1000) had the highest prevalence. Previous studies have not reported prevalence by nationality. It is known that prevalence of MetS varies by ethnicity [32] and given the diversity of the population in Qatar, the study provides essential information in this aspect, which was not available previously.

Prevention of the development of the first MetS component may have significant public health benefits as the presence of one component is predictive of the development of MetS [33]. While, public health strategies that are well known to be important for chronic disease prevention in general can substantially reduce the prevalence of metabolic syndrome [32]. The biggest challenges are identifying individuals with the greatest need for intervention due to their elevated risk for future disease and the population groups associated with the occurrence of MetS. The information reported in the study fill the gap in knowledge and is essential for the success of primary preventive measures of MetS in Qatar. Studies are needed to be establish which public health interventions are most effective in the population groups associated with high risk and occurrence of MetS.

The study has a number of strengths and limitations. Strengths include an up-to-date prevalence of MetS in primary care in Qatar. This provides a baseline for future longitudinal studies to monitor MetS and risk factors as well as in health planning and future strategies. The limitations are as follows: First, this was a cross-sectional study and provides a snapshot of the burden at a particular moment in time. Second, the study included only patients who were ≥ 18 years and those who attended a PHCC health centres in 2017; therefore, it is not nationally representative.

## Conclusions

The study provides essential epidemiological information required by decision makers. Although not nationally representative, this study is suggestive of a higher prevalence of MetS among a younger population, men and in Southern Asian, Northern African and Western Asian nationalities. Prevention, treatment and control of MetS is a public health problem in Qatar. More studies are needed to establish which public health interventions are likely to be effective in Qatar.

## Supplementary information

**Additional file 1.**

## Data Availability

All data generated or analysed during this study are included in this published article and its supplementary information files.

## Abbreviations

MetS: Metabolic Syndrome
T2DM: Type 2 Diabetes
PHCC: Primary Health Care Corporation
EMR: Electronic Medical Records
NCEP ATP III: National Cholesterol Education Program Adult Treatment Panel III
HDL: High-density lipoprotein
WHO: World Health Organisation
