# Supplementary material for: Prevalence of metabolic syndrome in primary health settings in Qatar: a cross sectional study
Source: BMC Public Health. 2020 May 3;20:611. doi: 10.1186/s12889-020-08609-5 (PMC7196222; doi:10.1186/s12889-020-08609-5)
Supplement: Supplementary file 1 — Additional file 1. [file 12889_2020_8609_MOESM1_ESM.docx]

**Table A: Metabolic Syndrome component definitions**

| **Components** | **NCEP ATP III definition** | **Study definition** | **Rationale** |
| --- | --- | --- | --- |
| Insulin resistance | Fasting blood sugar (FBS) over 100 mg/dl. | - Insulin Resistance (Serum Fasting Glucose>=100 mg/dl or HbA1c>=5.56) or - A diagnosis of T2DM | - HbA1c is an important marker ^1-3^ and enhances the detection of hyperglycemia for the diagnosis for MetS ^4^ |
| High Blood Pressure | Blood pressure over 130/85 mmHg | - Blood pressure over 130/85 mmHg or - A diagnosis of hypertension | Same definition used |
| Obesity | Waist circumference over 101.6 cm (40 inches) in men or 88.9 cm (35 inches) inwomen. | - Waist circumference over 102 cm (40 inches) in men and 94 centimetres (37 inches) in women   or   - Body Mass Index (BMI) >=30 kg/m2) | - Waist circumference are specific for each population therefore waist circumference for the Qatari population was used from a published study ^5^ - BMI value was shown by International Diabetes Federation to be a specific enough criteria to represent central obesity without the need for waist circumference measurements ^6^ |
| High serum triglycerides | Fasting triglyceride level over 1.7 mmol/L (150 mg/dl) | Fasting triglyceride level over 1.7 mmol/L (150 mg/dl) | Same definition used |
| Low serum HDL | Fasting high-density lipoprotein (HDL) cholesterol level <1.04 mmol/L (<40 mg/dl) in men or <1.3 mmol/L (<50 mg/dl) in women | Fasting high-density lipoprotein (HDL) cholesterol level <1.04 mmol/L (<40 mg/dl) in men or <1.3 mmol/L (<50 mg/dl) in women | Same definition used |

Table B: Missing data by Metabolic Syndrome Components

| **MetS criteria (Total population=421,283)** | **N** | **%** |
| --- | --- | --- |
| Obesity (Waist circumference over 102 centimetres in men and 94 centimetres in women or BMI>=30 kg/m2) | 135,859 | 32.2 |
| High Blood Pressure (over 130/85 mmHg or diagnosis of hypertension) | 13,808 | 3.3 |
| Low serum HDL (<1.04 mmol/L in men and <1.3 mmoL in women) | 237,711 | 56.4 |
| High serum triglycerides (>=1.7 mmol/L) | 236,612 | 56.2 |
| Insulin resistance (Serum Fasting Glucose>=100 mg/dl or HbA1c>=5.56 or diagnosed with T2DM) | 218,208 | 51.8 |

Table C: Available data by number of Metabolic Syndrome Components

| **Number of MetS criteria (Total population=421,283)** | **N** | **%** |
| --- | --- | --- |
| All 5 | 127,941 | 30.4 |
| At least 4 | 175,512 | 41.7 |
| At least 3 | 223,947 | 53.2 |
| At least 2 | 327,357 | 77.7 |
| At least 1 | 409,465 | 97.2 |

Table C: Country classification by region

| **Country Classification** | |
| --- | --- |
| **Region** | **Country** |
| Northern Africa | \| Algeria \| \| --- \| \| Egypt \| \| Libya \| \| Morocco \| \| Sudan \| \| Tunisia \| \| Western Sahara \| |
| Sub-Saharan Africa | \| British Indian Ocean Territory \| \| --- \| \| Burundi \| \| Comoros \| \| Djibouti \| \| Eritrea \| \| Ethiopia \| \| French Southern Territories \| \| Kenya \| \| Madagascar \| \| Malawi \| \| Mauritius \| \| Mayotte \| \| Mozambique \| \| Réunion \| \| Rwanda \| \| Seychelles \| \| Somalia \| \| South Sudan \| \| Uganda \| \| United Republic of Tanzania \| \| Zambia \| \| Zimbabwe \| \| Angola \| \| Cameroon \| \| Central African Republic \| \| Chad \| \| Congo \| \| Democratic Republic of the Congo \| \| Equatorial Guinea \| \| Gabon \| \| Sao Tome and Principe \| \| Botswana \| \| Eswatini \| \| Lesotho \| \| Namibia \| \| South Africa \| \| Benin \| \| Burkina Faso \| \| Cabo Verde \| \| Côte d’Ivoire \| \| Gambia \| \| Ghana \| \| Guinea \| \| Guinea-Bissau \| \| Liberia \| \| Mali \| \| Mauritania \| \| Niger \| \| Nigeria \| \| Saint Helena \| \| Senegal \| \| Sierra Leone \| \| Togo \| |
| Latin America and the Caribbean | \| Anguilla \| \| --- \| \| Antigua and Barbuda \| \| Aruba \| \| Bahamas \| \| Barbados \| \| Bonaire, Sint Eustatius and Saba \| \| British Virgin Islands \| \| Cayman Islands \| \| Cuba \| \| Curaçao \| \| Dominica \| \| Dominican Republic \| \| Grenada \| \| Guadeloupe \| \| Haiti \| \| Jamaica \| \| Martinique \| \| Montserrat \| \| Puerto Rico \| \| Saint Barthélemy \| \| Saint Kitts and Nevis \| \| Saint Lucia \| \| Saint Martin (French Part) \| \| Saint Vincent and the Grenadines \| \| Sint Maarten (Dutch part) \| \| Trinidad and Tobago \| \| Turks and Caicos Islands \| \| United States Virgin Islands \| \| Belize \| \| Costa Rica \| \| El Salvador \| \| Guatemala \| \| Honduras \| \| Mexico \| \| Nicaragua \| \| Panama \| \| South America \| \| Argentina \| \| Bolivia (Plurinational State of) \| \| Bouvet Island \| \| Brazil \| \| Chile \| \| Colombia \| \| Ecuador \| \| Falkland Islands (Malvinas) \| \| French Guiana \| \| Guyana \| \| Paraguay \| \| Peru \| \| South Georgia and the South Sandwich Islands \| \| Suriname \| \| Uruguay \| \| Venezuela (Bolivarian Republic of) \| |
| Northern America | \| Bermuda \| \| --- \| \| Canada \| \| Greenland \| \| Saint Pierre and Miquelon \| \| United States of America \| \| Antarctica \| |
| Eastern-Central Asia | \| Kazakhstan \| \| --- \| \| Kyrgyzstan \| \| Tajikistan \| \| Turkmenistan \| \| Uzbekistan \| \| China \| \| China, Hong Kong Special Administrative Region \| \| China, Macao Special Administrative Region \| \| Democratic People's Republic of Korea \| \| Japan \| \| Mongolia \| \| Republic of Korea \| |
| South-eastern Asia | \| Brunei Darussalam \| \| --- \| \| Cambodia \| \| Indonesia \| \| Lao People's Democratic Republic \| \| Malaysia \| \| Myanmar \| \| Philippines \| \| Singapore \| \| Thailand \| \| Timor-Leste \| \| Viet Nam \| |
| Southern Asia | \| Afghanistan \| \| --- \| \| Bangladesh \| \| Bhutan \| \| India \| \| Iran (Islamic Republic of) \| \| Maldives \| \| Nepal \| \| Pakistan \| \| Sri Lanka \| |
| Western Asia (excluding Qatar) | \| Armenia \| \| --- \| \| Azerbaijan \| \| Bahrain \| \| Cyprus \| \| Georgia \| \| Iraq \| \| Israel \| \| Jordan \| \| Kuwait \| \| Lebanon \| \| Oman \| \| Saudi Arabia \| \| State of Palestine \| \| Syrian Arab Republic \| \| Turkey \| \| United Arab Emirates \| \| Yemen \| |
| Eastern Europe | \| Belarus \| \| --- \| \| Bulgaria \| \| Czechia \| \| Hungary \| \| Poland \| \| Republic of Moldova \| \| Romania \| \| Russian Federation \| \| Slovakia \| \| Ukraine \| |
| Northern Europe | \| Åland Islands \| \| --- \| \| Channel Islands \| \| Guernsey \| \| Jersey \| \| Sark \| \| Denmark \| \| Estonia \| \| Faroe Islands \| \| Finland \| \| Iceland \| \| Ireland \| \| Isle of Man \| \| Latvia \| \| Lithuania \| \| Norway \| \| Svalbard and Jan Mayen Islands \| \| Sweden \| \| United Kingdom of Great Britain and Northern Ireland \| |
| Southern Europe | \| Albania \| \| --- \| \| Andorra \| \| Bosnia and Herzegovina \| \| Croatia \| \| Gibraltar \| \| Greece \| \| Holy See \| \| Italy \| \| Malta \| \| Montenegro \| \| Portugal \| \| San Marino \| \| Serbia \| \| Slovenia \| \| Spain \| \| The former Yugoslav Republic of Macedonia \| |
| Western Europe | \| Austria \| \| --- \| \| Belgium \| \| France \| \| Germany \| \| Liechtenstein \| \| Luxembourg \| \| Monaco \| \| Netherlands \| \| Switzerland \| |
| Australasia | \| Australia \| \| --- \| \| Christmas Island \| \| Cocos (Keeling) Islands \| \| Heard Island and McDonald Islands \| \| New Zealand \| \| Norfolk Island \| \| Fiji \| \| New Caledonia \| \| Papua New Guinea \| \| Solomon Islands \| \| Vanuatu \| \| Guam \| \| Kiribati \| \| Marshall Islands \| \| Micronesia (Federated States of) \| \| Nauru \| \| Northern Mariana Islands \| \| Palau \| \| United States Minor Outlying Islands \| \| American Samoa \| \| Cook Islands \| \| French Polynesia \| \| Niue \| \| Pitcairn \| \| Samoa \| \| Tokelau \| \| Tonga \| \| Tuvalu \| \| Wallis and Futuna Islands \| |

***References***

1. Osei K, Rhinesmith S, Gaillard T, Schuster D. Is glycosylated hemoglobin A1c a surrogate for metabolic syndrome in nondiabetic, first-degree relatives of African-American patients with type 2 diabetes? *J Clin Endocrinol Metab* 2003; 88: 4596-601.
2. Ong KL, Tso AW, Lam KS, Cherny SS, Sham PC, Cheung BM. Using glycosylated hemoglobin to define the metabolic syndrome in United States adults. *Diabetes Care* 2010; 33: 1856-8.
3. Lorenzo C, Wagenknecht LE, Hanley AJ, Rewers MJ, Karter AJ, Haffner SM. A1C between 5.7 and 6.4% as a marker for identifying pre-diabetes, insulin sensitivity and secretion, and cardiovascular risk factors: the Insulin Resistance Atherosclerosis Study (IRAS). *Diabetes Care* 2010; 33: 2104-9.
4. Siu P M and Yuen Q S. Supplementary use of HbA1c as hyperglycemic criterion to detect metabolic syndrome. *Diabetology & Metabolic Syndrome* 2014, 6: 119
5. Al-Thani MH, Al-Thani AAM, Cheema S, et al. Prevalence and determinants of metabolic syndrome in Qatar: results from a national Health survey. *BMJ Open* 2016;6:e009514.
6. Alberti KG, Eckel RH, Grundy SM, et al. International Diabetes Federation Task Force on Epidemiology and Prevention; National Heart, Lung, and Blood Institute; American Heart Association; World Heart Federation; International Atherosclerosis Society; International Association for the Study of Obesity. Harmonizing the metabolic syndrome: a joint interim statement of the International Diabetes Federation Task Force on Epidemiology and Prevention; National Heart, Lung, and Blood Institute; American Heart Association; World Heart Federation; International Atherosclerosis Society; and International Association for the Study of Obesity. *Circulation* 2009;120:1640–5.
